# Pathogenic ecological characteristics of PCV2 in large-scale pig farms in China affected by African swine fever in the surroundings from 2018 to 2021

**DOI:** 10.3389/fmicb.2022.1013617

**Published:** 2023-01-04

**Authors:** Chunqi Li, Guoping Liu, Ke Tong, Yan Wang, Tong Li, Xu Tan, Jie Yang, Xiaolin Yang, Liwei Guo, Jianguo Zeng

**Affiliations:** ^1^College of Animal Science, Yangtze University, Jingzhou, China; ^2^Hubei Institute of Cross Biological Health Industry Technology, Jingzhou, China; ^3^Hunan Key Laboratory of Traditional Chinese Veterinary Medicine, Hunan Agricultural University, Changsha, Hunan, China

**Keywords:** PCV2, PCV3, dual nested PCR, molecular epidemiology, positive rate of blood samples, excretion rate for PCV

## Abstract

Porcine circovirus type 2 (PCV2) has been identified as the causal agent of postweaning multisystemic wasting syndrome (PMWS), an economically important multifactorial disease of the swine industry worldwide. This research designed a dual nested polymerase chain reaction (PCR) detection method to simultaneously monitor porcine circovirus type 2 (PCV2) and PCV3. The limit of detection (LoD) of sensitivity for PCV2 and PCV3 was ten copies/mL for both viruses. There was no cross-reaction with any other porcine pathogens tested and no non-specific amplification. The coincidence and repetition rates were both 100%. Through the systematic and clinical sampling, 15,130 samples collected from 30 large-scale pig farms in eight provinces in China (including Hubei, Hunan, Henan, Jiangxi, Shanxi, Guangdong, Hainan, and Heilongjiang) were subjected to early warning surveillance and/or clinical diagnosis. These results revealed that the overall positive rates of PCV3 and PCV2 were 0 and 28.29%, respectively, with the lowest level recorded in Jiangxi province. The highest carrying rate was observed in Hainan province. Pigs at different ages displayed varying carrying rates for PCV: fattening pigs and gilts had the highest and the lowest carrying rates for PCV, respectively. In addition, the excretion rates for PCV of colostrum, semen, and nasal, anal, and vulval swabs were tested. The colostrum, anal swabs, and semen had higher excretion rates for PCV; these were followed by the vulval and nasal swabs that had excretion rates for PCV. Furthermore, a high blood virus-carrying rate was detected in moribund pigs, especially in pigs with fever and red skin. As to the virus-carrying rate in the pig organs received from clinical necropsy, the highest rate was found in placental tissue, followed by the kidneys, and the virus also was detected in lymphoid organs, liver, stomach, and intestines. The PCV2-positive samples were sequenced to reveal the molecular epidemic dynamics of PCV2. The results indicated four major branches, namely, PCV2a, PCV2b, PCV2c, and PCV2d, concerning PCV2 molecular epidemiology in China, with PCV2a, PCV2b, and PCV2d dominating. In conclusion, the results obtained in this study elucidated the molecular epidemiology, transmission, and positive blood samples of PCV and provided new ideas for developing comprehensive PCV control technologies to begin eliminating the disease caused by PCV by cleaning pig farms.

## 1. Introduction

Porcine circovirus (PCV), a member of the Circoviridae family, is one of the smallest DNA viruses discovered in vertebrates to date (Tischer et al., 1982). It has three serotypes: PCV1, PCV2, and the newly reported PCV3 (Opriessnig et al., 2007; Palinski et al., 2017). PCV1, a contaminant from porcine kidney cell lines, is apathogenic in pigs but can generate serum antibodies (Magar et al., 2000). PCV2 is pathogenic and primarily produces immunosuppression in animals. PCV2 is one of the world’s most severe infectious swine diseases. It is the major pathogen involved in postweaning multisystemic wasting syndrome (PMWS) (Palinski et al., 2017) and porcine dermatitis and nephropathy syndrome (PDNS; Allan et al., 2000). PCV2 can infect pigs at any age, but five to 16-week-old pigs are more prone to PMWS (Segales et al., 2005), while fattening pigs (16–22 weeks old) often suffer from a respiratory disease syndrome.

The emerging PCV3 serotype is associated with porcine dermatitis, nephrotic syndrome, and reproductive failure, as well as cardiac and multisystem inflammation, but its pathogenic mechanism remains unclear. PCV3 was first reported in the United States and was related to porcine dermatitis, nephrotic syndrome, and abortion in sows. Sows infected with PCV3 exhibit signs of anorexia, multifocal papules and spots, and superficial dermatitis, in addition to decreased production performance (Opriessnig et al., 2007). Infected pregnant sows suffer reproductive disorders, giving birth to weak, stillborn, and mummified fetuses or weak piglets that die in acutely severe cases, with fetuses of different gestational ages at risk of abortion (Sanchez et al., 2001). Due to the low viral load observed in pigs subclinically infected with PCV2, traditional polymerase chain reactions (PCR) yield poor sensitivity, and infections are often missed. Currently, research related to PCV3 is still in its infancy, and effective prevention and control measures are lacking. Therefore, the pathogenic ecology of PCV3 should be systematically explored as a matter of urgency.

Since 3 August, 2018, when the first case of African swine fever was reported in Shenyang province, China, this viral disease has been successively reported in all provinces and posed a grave threat to the pig industry in China (Ge et al., 2018). To control African swine fever, an effective and readily accessible comprehensive technical system has been established in China by pooling information from across the country and beyond. The system includes management, production, and technical aspects that provide early warning, upgrade of facility biosecurity, strengthening of biosecurity and management, control of risks, adjustment of control modes for major diseases at pig farms, and rapid decontamination techniques in emergency situations.

To respond to African swine fever, basic vaccines, including the vaccine of PCV, have almost all ceased on many large-scale farms to control the risk of possibly importing or cross-infection of the disease due to personnel contact with pigs. Thus, the once well-controlled PCV disease has made a comeback, and whether the impact of circovirus on pigs has been aggravated because of this, and whether the molecular characteristics of circoviruses have changed are all presently unknown due to a lack of published literature. Therefore, systematic sampling and evaluation of PCV backgrounds should be conducted, and the positive blood samples status of pigs, distribution of PCV in organs, excretion for PCV of pigs, and molecular epidemic characteristics of the pathogen need to be investigated.

Considering the current situation, a dual nested PCR protocol with excellent sensitivity, good specificity, and the ability to sequence positive samples efficiently while monitoring both PCV2 and PCV3 was constructed in this study. The dual nested PCR protocol was utilized to systematically assess samples from 30 large-scale pig farms, each containing more than 500 sows, in eight provinces in China to clarify the epidemic status, transmission characteristics, and molecular epidemic characteristics of PCV diseases. This study provided systematic support for controlling and eliminating the diseases caused by PCV due to the effects of African swine fever.

## 2. Materials and methods

### 2.1. Nested PCR establishment

#### 2.1.1. Viruses and plasmids

The pathogens PCV2, classical swine fever virus (CSFV), porcine parvovirus (PPV), pseudorabies virus (PRV), and porcine respiratory and reproductive syndrome virus (PRRSV) were identified and maintained in our laboratory. PCV3 and the other plasmids used in this study were constructed and preserved within the severe disease early warning and decontamination team laboratory. A spectrophotometer was used to measure the standard plasmid concentrations. The copy number was calculated based on the following formula: copies/mL = (6.02 × 10(23) copies/mol) × (concentration)/(MW g/mol; Trottier et al., 2002).

#### 2.1.2. Main reagents and primers

Commercially available premix Taq MIX and nucleic acid extraction kits (Tiangen, China), DL2000 Marker and nucleic acid dyes (Solarbio, China), PCV2 enzyme-linked immunosorbent assay (ELISA) detection kits (MEDIAN, Korea), Quantitative PCR (qPCR) detection kits for PCV2 (IDEXX, United States) and PCV3 (IDEXX, USA), and African Swine fever virus real-time PCR detection kit (NECVB, Harbin, China) were acquired. Two pairs of specific primers for PCV 2 and two pairs of specific primers for PCV 3 were designed based on the nucleotide sequence of the Cap protein ORF 2 using Oligo 6.0 and Primer 5.0 software after referring to PCV 2 and PCV 3 gene sequences and the relevant literature. They were synthesized by Sangon Biotech (Shanghai, China) and stored at −20°C. The sequences are shown in Supplementary Table S1.

#### 2.1.3. Optimization of dual nested PCR

Between 0.5 ~ 1.5 μl of primers in 0.5 μl increments were used. The optimal reaction system and optimal reaction program were selected, and two rounds of PCR reactions were performed. The 25 μl PCR reaction contained 12.5 μl of 2 × Taq Mix, 1 μl of PCV2 and PCV3 upstream and downstream primers (10 μmol/l), respectively (the first round was O-F, O-R as Outside-Forward, Outside-Reverse; the second round was I-F, I-R as Inside-Forward, Inside-Reverse), 8.5 μl of ddH2O, and 1 μl of the template, and the first amplification product was used as the template for the second reaction. The amplified products from the two reactions were detected following electrophoresis using 1% agarose gels.

#### 2.1.4. Specificity and sensitivity tests for the dual nested PCR and its coincidence rate

Using the genomes of CSFV, PPV, PRV, PRRSV, *Escherichia coli*, and *Staphylococcus aureus* as templates and recombinant plasmids as controls, the four pairs of primers, PCV2-O-F and PCV2-O-R, PCV3-O-F and PCV3-O-R, PCV2-I-F and PCV2-I-R, and PCV3-I-F, and PCV3-I-R, used in the nested PCR were tested for specificity. In addition, the concentration of the PCV2 genome-wide positive control and recombinant plasmid pMD20-T-PCV3 was determined, followed by a copy number calculation. Next, the plasmids were diluted 10-fold, and the plasmids in each dilution were used as the template for the first PCR amplification with the primers, PCV2-O-F, PCV2-O-R, PCV3-O-F, and PCV3-O-R. The resulting products were used as the template of the corresponding gradient for the second PCR amplification using primers PCV2-I-F, PCV2-I-R, PCV3-I-F, and PCV3-I-R. The products from both the first and second amplification were detected using 1.0% agarose gels, and the smallest detectable number of copies was calculated. On this basis, the PCV2 genome-wide positive controls with copy numbers of 10^5^, 10^4^, 10^3^, 10^2^, 10^1^, and 10^0^, and PCV3 plasmid controls and the same batch of clinical samples collected in Hubei were tested using the established nested PCR, quantitative fluorescence PCR, and conventional PCR. And ELISA was used to test these clinical samples. The test results were used to validate the coincidence rate of the nested PCR.

### 2.2. Detection of African swine fever virus in the environment around pig farms

From 2018 to 2021, we collected 2056 environmental samples within 3 kilometers around 30 pig farms in eight provinces on a quarterly basis, including soil, water, vehicles, people, and articles. The collected environmental samples were tested with the African Swine fever virus real-time PCR detection kit (NECVB, Harbin, China) according to the manufacturer’s instructions.

### 2.3. Application of dual nested PCR

**Figure 1:**
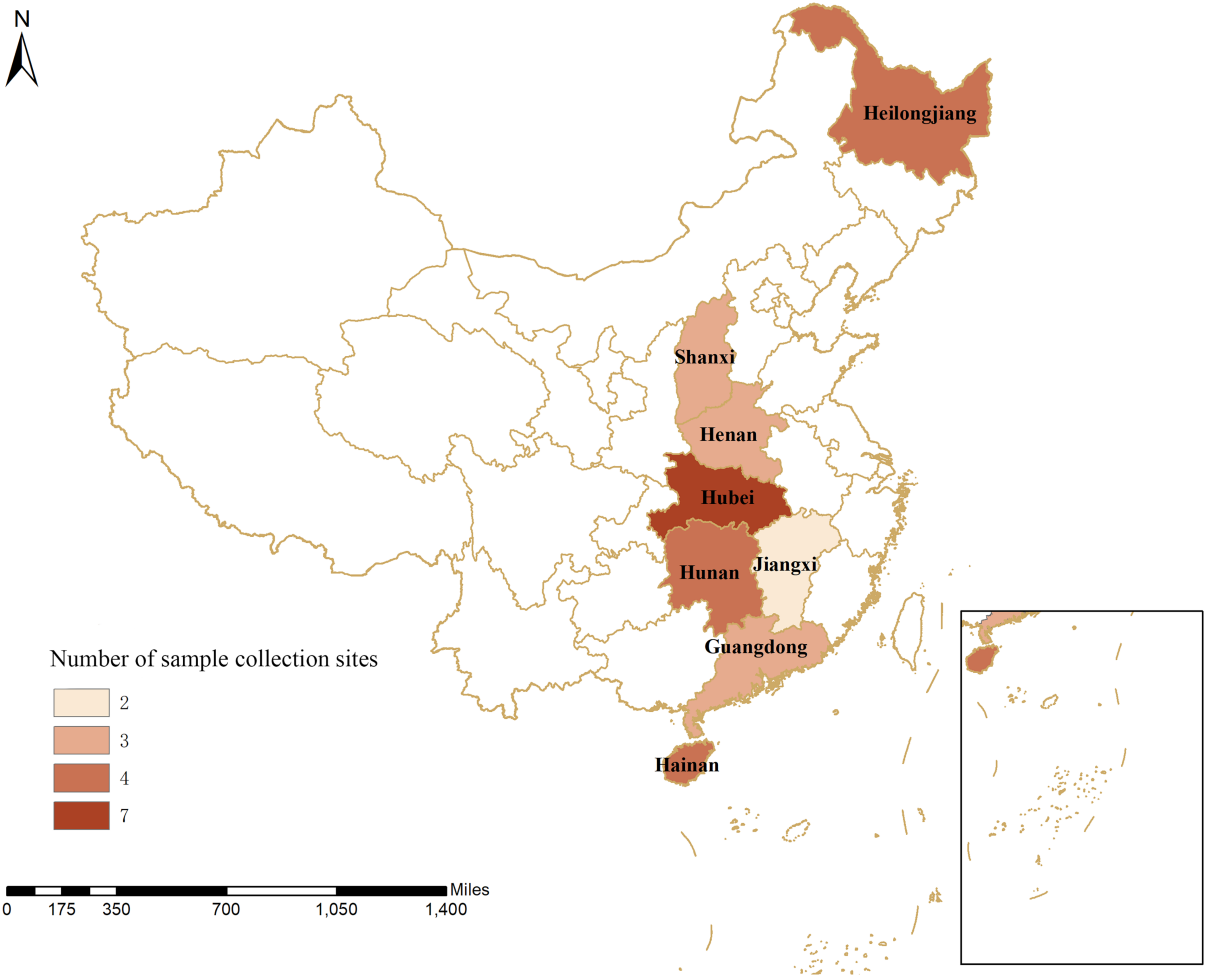
Geographical distribution of sample collection from 30 pig farms in eight provinces in China. The numbers show the distribution of pigs on the farms within the provinces in China.

The dual nested PCR established in this study was used for clinical testing of 15,130 clinical samples from 30 large-scale pig farms (breeding stock >500 sows) in eight provinces, including Hubei, Hunan, Henan, Jiangxi, Shanxi, Guangdong, Hainan, and Heilongjiang provinces (Figure 1) or during the evaluation of early warning surveillance of systematic sampling as per the sampling procedures. In addition, an analysis was conducted on the prevalence and characteristics of PCV, specifically, the virus-carrying rate in the blood and organs, excretion for PCV in semen, colostrum, and saliva, as well as nasal, anal, and vulva swabs, and genetic characteristics of the pathogen, in some areas of China.

**Figure 2:**
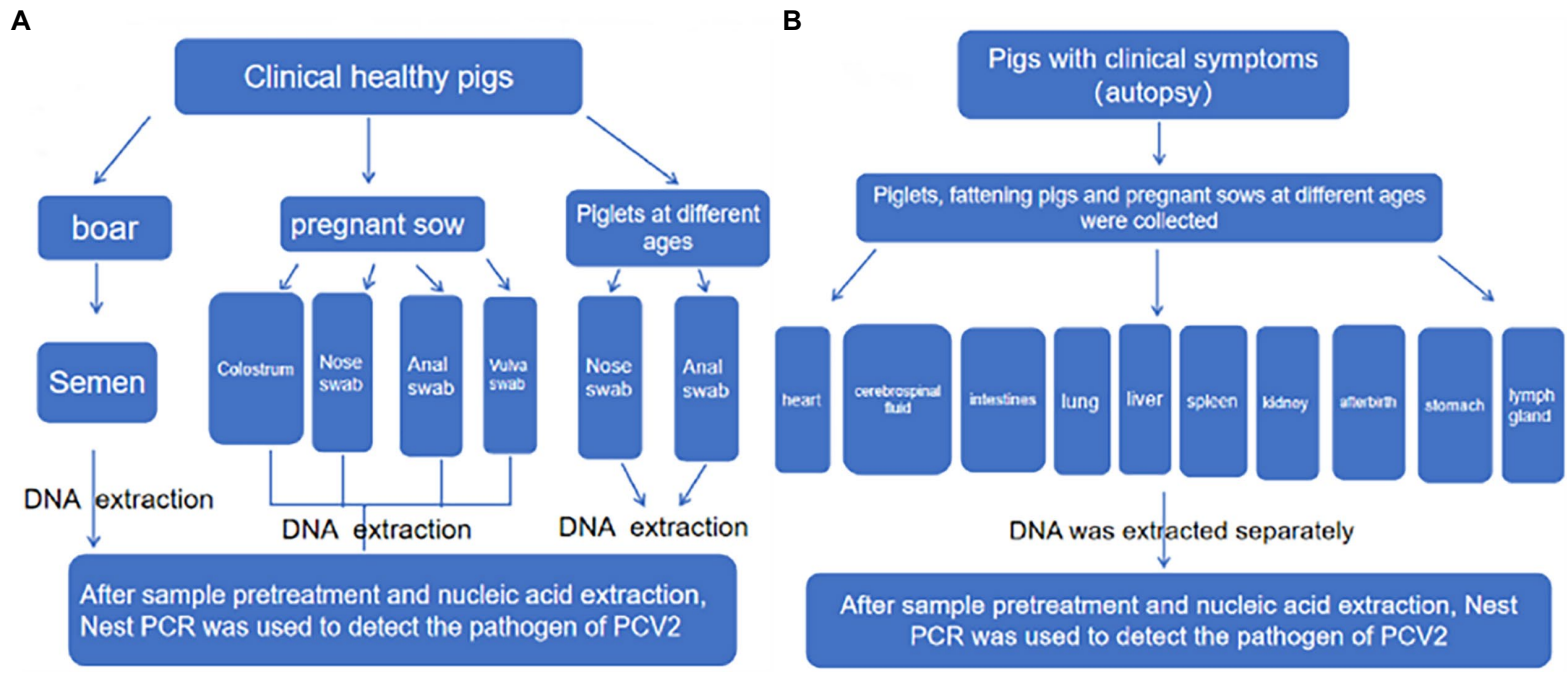
Sampling scheme diagram. **(A)** Clinically healthy pigs. **(B)** Piglets at different ages.

An epidemiological survey was performed on pigs without apparent clinical signs (Figure 2). Specifically, semen from boars and nasal, anal, and vulval swabs and colostrum from pregnant sows were systematically collected in a standardized manner. DNA was obtained from samples taken from the nose, anus, and vulva from offspring at different ages, including 30–50 days, 50–70 days, 70–90 days, and over 90 days. A total of 12,691 samples were obtained throughout the study. Furthermore, organ examination was carried out on pigs with suspected clinical signs that included anorexia, redness of the conjunctiva, pale skin, lethargy, and skin disease, as well as sows with reproductive disorders and weak piglets with congenital tremors. Samples were harvested from the heart, cerebrospinal fluid, intestines, lung, liver, spleen, kidney, afterbirth, stomach, and lymph nodes. A total of 2,194 samples were obtained *via* necropsy from 300 pigs.

Samples were collected using a standardized protocol and stored in a closed low-temperature styrofoam box at 4°C, then transported to the laboratory within 1–2 h for subsequent processing. The swabs (semen or colostrum) were placed in a 5 ml Eppendorf (Ep) tube and soaked in an appropriate amount of normal saline, followed by 30 s of vortexing. The supernatant was retrieved, placed on a shaker for 30 min, transferred to a 2 ml Ep tube, completely homogenized, and centrifuged. The supernatant was harvested for subsequent DNA extraction. Fresh tissues were collected from the junction between healthy and diseased sites. Then, blood and connective tissues were removed with sterile medical gauze, and the samples were cut into small pieces on ice. The tissue pieces were transferred into a pre-cooled homogenizer, mixed with a homogenization buffer composed of 0.25 mmol/l sucrose, 25 mmol/l Tris–HCl (pH 7.5), 25 mmol/l NaCl, and 25 mmol/l MgCl_2_ at 10 ml/g. The homogenate was placed on a shaker for 30 min and then homogenized. Following centrifugation at 12,000 r/min for 5 min, the supernatant was collected. All positive samples were sent to Wuhan Sangon for sequencing.

## 3. Results

### 3.1. Establishment of dual nested PCR

#### 3.1.1. Optimization of the reaction conditions

The optimized PCR conditions, including annealing temperatures (56°C and 58°C) and template and primer volumes for the four pairs of primers for dual nested PCR, were used with the specific dual nested PCR reaction conditions. After optimizing the dual nested PCR conditions for PCV2 and PCV3, there were distinct target bands that did not produce heterobands. The optimal conditions for the dual nested PCR are listed in Supplementary Table S2.

#### 3.1.2. Specificity test

The genomes of CSFV, PPV, PRV, PRRSV, *Escherichia coli*, and *Staphylococcus aureus* were extracted as templates for the specificity test using the four pairs of primers (PCV2-O-F and PCV2-O-R, PCV3-O-F and PCV3-O-R, PCV2-I-F and PCV2-I-R, and PCV3-I-F and PCV3-I-R), which were used in the dual nested PCR. As shown in Supplementary Figure S1(A) PCV2 and PCV3 exhibited no cross-reactions with these other viruses. No heterobands or dimers were present, indicating that the four pairs of primers had good specificity.

#### 3.1.3. Sensitivity test (dual nested PCR)

Different gradients of the PCV2 whole-genome template and PCV3 recombinant plasmid were amplified using the specific primers, and the virus concentrations were detected concomitantly. The limit of detection (LoD) for PCV2 and PCV3 was ten copies/mL, and the number of copies of PCV2 and PCV3 dilution gradients was 1 × 10^9^, 1 × 10^8^, 1 × 10^7^, 1 × 10^6^, 1 × 10^5^, 1 × 10^4^, 1 × 10^3^, 1 × 10^2^, 1 × 10^1^, and 1 × 10^0^, in sequence. As shown in Supplementary Figure S1(B), the LoD of PCV2 and PCV3 detected after two amplifications using the dual nested PCR was ten copies/mL, two to three orders of magnitude higher than that obtained by the current detection method. Regarding interference from other pathogens, the amplified bands exhibited no changes (Supplementary Figure S1(C)).

#### 3.1.4. Coincidence rate and repeatability test

To confirm the coincidence rate of the dual nested PCR, the same batch of clinical samples (including 150 serum samples: 103 PCV2-positive samples and 0 PCV3-positive samples) were tested separately using the dual nested PCR established in this study, a commercial ELISA kit for PCV2, and a fluorescence quantitative PCR detection kit for PCV2 and PCV3 (IDEXX). The test results were compared (Supplementary Table S3) to validate the coincidence rate of the dual nested PCR. Each sample was analyzed in triplicate, and the results were consistent between the three runs, with a repetition rate of 100%. The results demonstrated that the coincidence rate of the dual nested PCR was 100%, and the LoD for PCV2 and PCV3 was ten copies/mL. In addition, there were no false positives or false negatives indicated by the dual nested PCR, according to the sequencing results. In the other conventional methods, the coincidence rate was low. The LoD for PCV2 was 1 × 10^5^–1 × 10^1^, and no PCV3 was detected. The LoD for the positive plasmids also was ten copies/mL. In addition to the low coincidence rate, virus-negative samples were more likely to test as false positive *via* ELISA and qPCR, which could lead to an incorrect diagnosis.

### 3.2. Correlation analysis between ASFV and PCV2

The test results showed that 510 samples were positive for ASFV. We used JMP software to analyze the positive rate of ASFV and the positive rate of the PCV2, and the partial correlation reached 0.6, indicating that ASFV and the PCV2 had better correlation (Supplementary Table S4).

### 3.3. Epidemic research based on dual nested PCR

#### 3.3.1. Prevalence of PCV2 in eight provinces of China

A total of 15,130 clinical samples, including whole blood, colostrum, and semen, as well as nasal, vulval, and anal swabs, were collected from pigs at different ages from eight provinces (Table 1). PCV2 showed a positive rate of 25.61% (599/2,344), 33.64% (529/1,801), 16.79% (391/2,329), 8.31% (133/1,600), 20.30% (307/1,512), 22.63% (415/1,834), 42.36% (726/1,799), and 26.22% (501/1,911) in Hubei, Hunan, Henan, Jiangxi, Shanxi, Guangdong, Hainan, and Heilongjiang provinces, respectively. Thus, the positive rate of PCV2 ranged from 8.31 to 42.36%, averaging 24.04% (3,637/15,130), which was highest in Hainan (42.36%, 762/1,799), followed by Hunan, Heilongjiang, Guangdong, Hubei, Shanxi, and Henan provinces, with Jiangxi province exhibiting the lowest rate (8.31%, 133/1,600). The detection results of the mixed infection indicated that the single infection rate of PCV2 was relatively low (3.83%), while the remaining samples exhibited a double or triple mixed infection at 31.55 and 10.61% for all samples, respectively. In the double infection samples, PCV2 + PRRSV, PCV2 + PRV, and PCV2 + PPV were the predominant types observed, with positive rates of 21.28, 6.1, and 4.17% of all samples, respectively, while PCV2 + PRRSV+PRV and PCV2 + PRRSV+PPV were most prevalent in the triple infection samples, with positive rates of 7.06 and 3.55% of all samples, respectively. Other types of mixed infection were rare. This indicated that in some regions of the eight provinces in China, PCV2 was widely prevalent in large-scale pig farms, while the positive rates of mixed infection occurred to varying extents and indicated significant differences (*p* < 0.05; Table 1).

**Table 1:** Prevalence of Porcine Circovirus type 2 in surveillance samples from eight provinces.

Province	Tested samples	Positive samples	Positive rate(%)	The infection type	Infection pattern	Infection rate(%)
Hubei	2,344	599	25.61% (599/2344)	A single infection	PCV2	3.83% (579/15130) ab
					PCV3	0% (0/15130) c
Hunan	1801	529	33.64% (529/1801)	Double infection	PCV2 + PRRSV	21.28% (3,219/15130) g
Henan	2,329	391	16.79% (391/2329)		PCV2 + PRV	6.10% (923/15130) h
Jiangxi	1,600	133	8.31% (133/1600)		PCV2 + PPV	4.17% (631/15130) b
Shanxi	1,512	307	20.30% (307/1512)	Triple infection	PCV2 + PRRSV+PRV	7.06% (1,068/15130) i
Guangdong	1834	415	22.63% (415/1834)		PCV2 + PRRSV+PPV	3.55% (537/15130) a
Hainan	1799	762	42.36% (762/1799)		PCV2 + PRV + PPV	0.23% (35/15130) j
Heilongjiang	1911	501	26.22% (501/1911)	Multiple infection	PCV2 + PRRSV+PRV + PPV	0.09% (13/15130) k

15,130 clinical samples were collected from eight provinces. Blood and colostrum as well as nasal, vulva, and anal swabs were collected from pigs of different ages and stages of development. The test results showed single infections, double infections, and multiple mixed infections. The SPSS 24.0 chi-square test was used to analyze the differences between the detection results in samples with mixed infections from different regions and from other diseases. *p* < 0.05 was considered significant.

#### 3.3.2. PCV2 Infection in pigs at different ages

**Figure 3:**
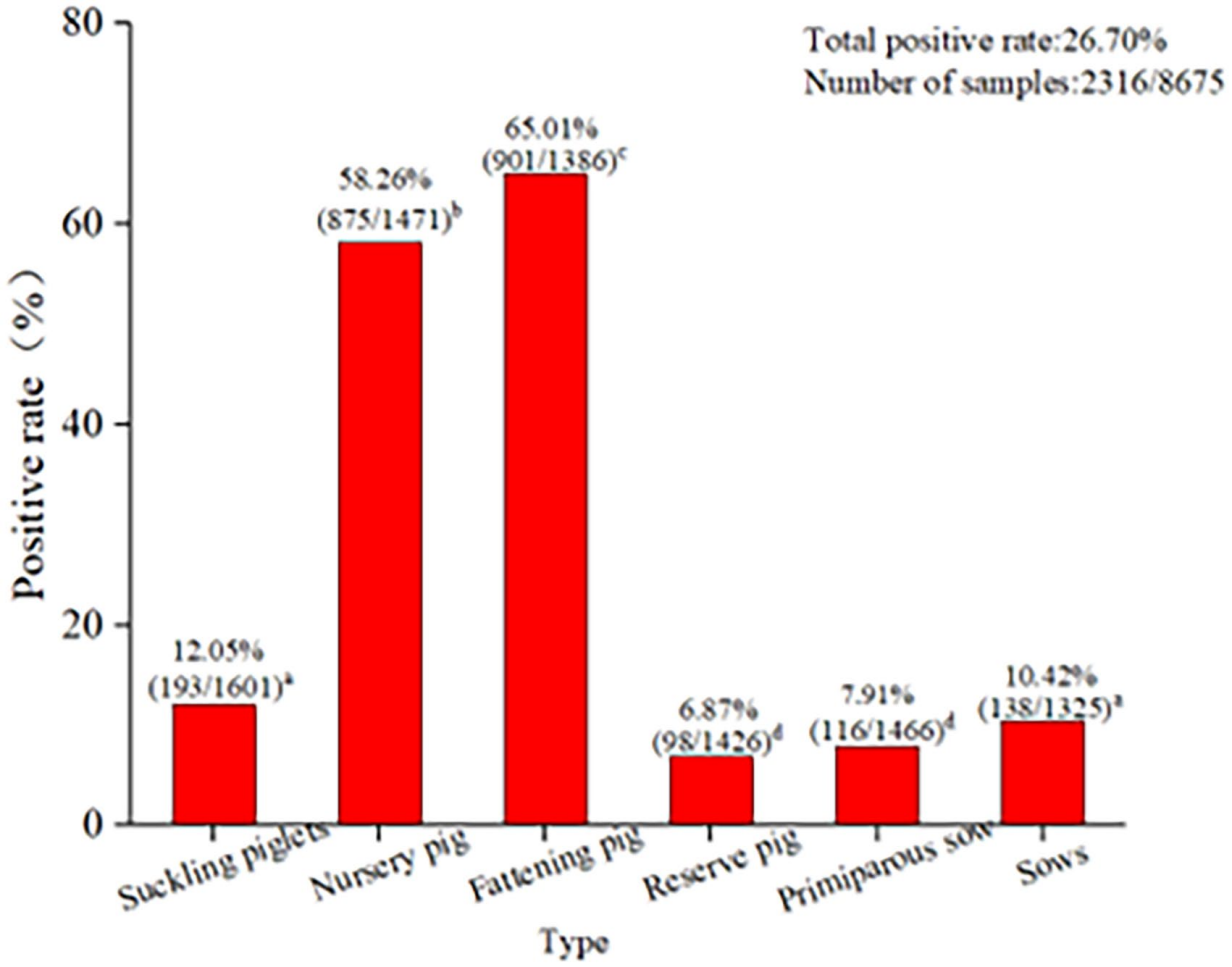
PCV2 infection in pigs at different ages in eight provinces. The PCV2 infection in pigs at different ages indicated that PCV2 was able to infect pigs of all ages. The positive rate of PCV2 in finishing pigs (65.01%) was the highest, followed by nursing pigs (58.26%) and lactating pigs (12.05%). The positive rates of economic sows, gilts, and primiparous sows were 10.42, 6.87, and 7.91%, respectively. The test results for PCV2 infection in pigs at different ages in the eight provinces were analyzed using the SPSS 24.0 chi-square test. *p* < 0.05 was considered significant.

Among the 8,675 blood samples from the eight provinces, PCV2 infection was detected in pigs at different ages, indicating that PCV2 could infect pigs at all ages (*p* < 0.05, Figure 3). Specifically, the PCV2-positive rate was highest in fattening pigs (65.01%), followed by nursing pigs (58.26%), then suckling piglets (12.05%), while it was relatively low in multiparous sows (10.42%), primiparous sows (7.91%), and gilts (6.87%; Figure 3). These results demonstrated that infection occurred in pigs at different ages, with significant differences in the positive rates observed. The highest infection rate was detected in fattening pigs aged over 130 days old.

#### 3.3.3. PCV2 Infection in pigs with different clinical signs

Blood samples collected from pigs with different clinical signs from the pig farms were sent for PCV2 pathogenic surveillance (Table 2). The results showed that the detection rate for positive blood samples was 27.81%. PCV2 had a positive rate of 58.79% in the blood samples from nursing pigs and fattening pigs that presented with red and cyanotic skin, which was the highest positive rate detected in blood. Pregnant sows with lochia, premature delivery, and abortion ranked second for positive in blood (28.92%). Thereafter, the detection rates of positive blood samples were seen in moribund weak piglets among suckling piglets, nursing piglets, and sows with loss of appetite, moribund defective pigs among fattening pigs, and moribund nursing or suckling piglets with diarrhea at rates of 27.91, 22.09, 16.67, and 13.19%, respectively. Positive blood samples also were detected in a few moribund stiff piglets among nursing piglets or piglets that had died of encephalitis, with positive rates of 1.67 and 1.96%, respectively.

**Table 2:** Distribution of different viremias.

Sample Type	Age Stage	Clinical Signs	Tested samples	Positive samples	Positive rate
Whole blood	Suckling piglets	Weak piglet death	129	36	27.91%
	Suckling and nursing piglets	Piglet diarrhea	91	12	13.19%
	Nursing piglets	Piglets died of encephalitis	51	1	1.96%
	Nursing piglets	Dead stiff pig	60	1	1.67%
	Nursing and fattening pigs	Dead pigs with red and cyanotic skin	165	97	58.79%
	Fattening pigs	Residual pig death	54	9	16.67%
	Nursing piglets and sow	Emaciation and loss of appetite	86	19	22.09%
	Pregnant sows	Reproductive disorder	166	48	28.92%

Collection of blood from pigs with different clinical signs in pig farms for PCV2 etiological monitoring.

#### 3.3.4. PCV2 Infection In different tissues and organs of pigs

The samples that tested positive for PCV2 accounted for 19.43% of the 794 samples retrieved following necropsy (Table 3). PCV2 was detected in all the tissue and organ types submitted for inspection. The highest positive rate was present in afterbirth (in 41.67% of the infected animals with an afterbirth sample collected), followed by the kidney (34.43%), lymph nodes (31.58%), liver (23.94%), spleen (22.54%), heart (14.08%), intestines (13.64%), lung (12.68%), and stomach (8.45%). The examined blood samples exhibited a total positive rate for PCV2 of 20.41%. It should be noted that PCV2 also was detected in cerebrospinal fluid, with a positive rate of 11.11% (4/36).

**Table 3:** Distribution of PCV2 in different organs.

Organ Type	Number of samples	Positive number	Positive rate
Heart	71	10	14.08%
Cerebrospinal fluid	36	4	11.11%
Intestines	66	9	13.64%
Lung	71	9	12.68%
Liver	71	17	23.94%
Spleen	71	16	22.54%
Kidney	61	21	34.43%
Afterbirth	12	5	41.67%
Stomach	71	6	8.45%
Lymph gland	19	6	31.58%
Blood	245	50	20.41%

A total of 794 clinically suspected samples were necropsied, and the overall positive rate of PCV2 was 19.43%. PCV2 was detected in all tissue and organ types submitted for inspection.

#### 3.3.5. Excretion ways for PCV2

**Figure 4:**
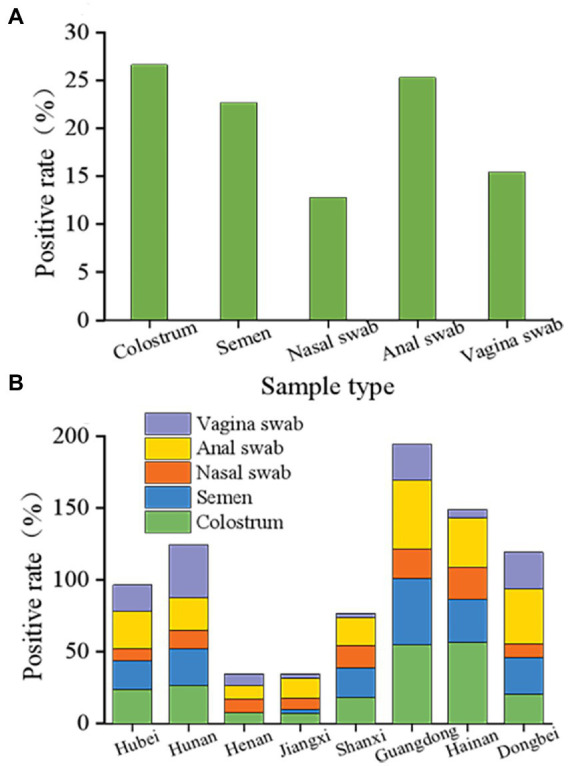
Overall status of porcine circovirus type 2 excretion. **(A)** Overall elimination of PCV2 in different organs of pigs in eight provinces. PCV2 is detoxified through colostrum, semen, nose, anus, and vulva and still showed a high positive rate following immunization with the ring vaccine. The positive rates of colostrum, semen, and anal swabs were similar, 26.65, 22.71, and 25.30%, respectively. Nasal swabs and vulvar swabs were recorded as 12.81 and 15.47%, respectively. **(B)** Different excretion pathways of pigs in the eight provinces. Among them, the positive range of colostrum was 7.35 to 56.69%, and the positive rate of colostrum from Jiangxi province was the lowest at 7.35%. The positive colostrum rate in Hainan province was the highest at 56.69%. The lowest semen-positive rate was 0 in Henan province, while the highest was observed in Guangdong province at 46.35%. The detection rates in the anus, nose, and vulva were 9.26 to 47.79%, 7.74 to 22.02%, and 2.67 to 36.84%, and the lowest detection rates were in the Henan, Shanxi, and Jiangxi provinces. The provinces with the highest detection rates were Guangdong, Hainan, and Hunan.

These results indicated that PCV2 could be passed through colostrum, semen, nose, anus, and vulva, and still showed a high positive rate. The positive rates observed in colostrum, semen, and anal swabs were relatively similar at 26.65, 22.71, and 25.30%, respectively (Figure 4A). However, the rates for the nasal and vulvar swabs were lower at 12.81 and 15.47%, respectively (Figure 4A). Although the way of excreting PCV2 in various provinces and regions was similar, notable differences were found in the excretion rate for PCV2 in the different growth stages of pigs. According to the statistical analysis (Figure 4B), the positive rates for colostrum were lowest in Jiangxi province (7.35%) and the highest in Hainan province (56.69%), and for semen, the lowest rate was in Henan province (0%) and the highest in Guangdong province (46.35%). The positive rates detected in the anus, nose, and vulva ranged from 9.26–47.79%, 7.74–22.02%, and 2.67–36.84%, respectively, with the lowest in Henan, Jiangxi, and Shanxi provinces and the highest in Guangdong, Hainan, and Hunan provinces.

#### 3.3.6. Sequence analysis of PCV2

The variation within the PCV2 Cap protein allowed changes in viral immunogenicity and cell tropism, resulting in differences in virulence. The analysis completed on the Cap amino acid sequences revealed several amino acid variations in the Cap protein (Table 4). Specifically, the PCV2 strains in the genotype PCV2a, PCV2b, PCV2c, and PCV2d harbored ten unique amino acid mutations, respectively. These amino acid loci were highly variable, and amino acids changed from hydrophobic to hydrophilic, with the potential to cause changes in the antigenicity of PCV strains.

**Table 4:** Amino acid substitutions in the putative Cap protein.

Classic strains	Position	PCV2a	PCV2b	PCV2c	PCV2d
PCV2a-HBxz (GenBank: FJ870968.1)	70(D)	G(2)/L(1)/T(1)	S(2)/L(1)/A(1)	*(2)	L(2)/S(1)/I(1)
	73(R)	N(3)/F(2)	H(2)/*p*(1)/D(1)	L(1)	R(1)/D(1)/H(1)/*F*(1)
	103(V)	A(9)	D(3)	A(2)	H(2)
PCV2b-ZD-1-CHN-2014-PCV2 (GenBank: KU041849.1)	106(W/F)	Q(9)	H(2)	Q(2)	H(2)
	112(T)	*p*(9)	K(3)	*p*(2)	*p*(6)
Pcv2c-DK1980PMWSfree (GenBank: EU148503.1)	115(D)	A(1)	A(1)	S(2)	A(2)
	145(S)	E(1)	E(4)	G(2)	E(2)
	178(N)	R(2)	R(4)	Q(2)	R(3)
Pcv2d-ChenZ-2-1 (GenBank: MH718995.1)	205(S)	A(9)	D(3)	A(2)	A(6)
	211(Y)	V(1)	V(4)	L(2)	V(3)

The positive products from the Hubei, Hunan, Henan, Jiangxi, Shanxi, Guangdong, Hainan, and Heilongjiang provinces were purified and sequenced. Twenty-four isolates with gene sequences were obtained and compared to the sequences from domestic reference strains accessed on MEGA 5.0. According to the phylogenetic comparative analysis, the homology (95.36 to 99.95%) was ascertained between isolates and the classical strains (Supplementary Figure S2(A)). The sequence homology of PCV2 varied in different regions and showed the most similarity between Guangdong01 and Henan02 (99.95% homology) and the largest difference between Henan03 and Hubei03 (95.36% homology). In addition, a homology of 95.46 to 99.98% was validated between the 24 isolates and foreign strains, which revealed the largest variation was between Shanxi03 and JX535286 (95.51% homology), and Hubei01 and KM245558.GX (95.46% homology).

Four genotypes were identified in the 24 isolates, including PCV2a (37.50%), PCV2b (29.17%), PCV2c (0.083%), and PCV2d (25.00%). The epidemic strains were distributed slightly differently in the various regions, with PCV2d (3/5) and PCV2b (2/5) present in Hubei province, PCV2c and PCV2d in Hunan province, PCV2a in Jiangxi and Heilongjiang provinces, PCV2b in Shanxi and Hainan provinces, PCV2a and PCV2d in Guangdong province, and PCV2a (3) in Henan province. As shown in Supplementary Figure S2(B), a phylogenetic tree of PCV2 was constructed, and the data indicated that the sequences were primarily divided into four branches, which belonged to reference strains, PCV2a, PCV2b, PCV2c, and PCV2d. Shanxi01 and Shanxi02 were similar to Hunan02, revealing close homology.

## 4. Discussion

### 4.1. The high sensitivity of dual nested PCR

In this study, four pairs of detection primers were designed *via* encoding the PCV2 and PCV3 Cap proteins and optimizing the conditions required for the analysis. The findings revealed that the dual nested PCR constructed showed good specificity when detecting PCV2 and PCV3, with repeatability and coincidence rates of 100%, and they showed no cross-reaction with CSFV, PPV, PRV, or PRRSV. The LoD for both PCV2 and PCV3 was ten copies/mL. Therefore, the detection method developed in this study exhibited a relatively high sensitivity compared to the methods in previous reports. The sensitivity of this assay was 2.9 copies for the PCV2 plasmid and 22.5 copies for the PCV3 plasmid (Li et al., 2018). In comparison, the detection limit for ddPCR was two copies/μL for PCV2 and 1 copy/μL for PCV3. The detection limits of another experimental method for PCV2 and PCV3 were ten and one copies/μL (Cao et al., 2021). Therefore, the present method provided favorable anti-interference, specificity, and repeatability and is applicable to clinical or normal diagnosis and detection, early warning, and surveillance of PCV2 and PCV3. Based on a comparative analysis, the previous methods are less sensitive than the current constructed method. The nested PCR could simultaneously detect PCV2 and PCV3 and promptly enabled gene sequencing to be performed following the presence of positive results. This enabled precise assessment of the epidemic situations relating to PCV2 and PCV3 in pig farms facing the current pressure from African swine fever, saving etiological confirmation time and reducing costs.

### 4.2. PCV prevalence under the pressure of African swine fever

Circovirus disease was once prevalent in large-scale pig farms in China (Shuai et al., 2007; Wang et al., 2009; Guo et al., 2019), but its incidence gradually declined with the implementation of prevention and control measures. Nevertheless, in order to prevent the infection of African swine fever virus, which first appeared in 2018, disease prevention and environmental management in China’s pig industry have been significantly improved. Since the infection with the African swine fever virus, some pig farms have not even performed basic immunization due to the need for safe production. In these cases, the large pig farming industry in China may face large-scale economic losses once the infection of circovirus disease appears.

Therefore, because epidemiological surveys on the infection and transmission of PCV remain inconclusive, it is critical to comprehensively and systematically explore the pathogenic ecological characteristics of PCV2 and PCV3 under the effect of African swine fever. In this study, the etiology of PCV in clinical samples sent from large-scale pig farms in several regions of China was detected *via* dual nested PCR. The results indicated no PCV3-positive samples (positive rate: 0%), which was inconsistent with the detection rate of PCV3 (2.96 to 37.96%) in various domestic regions previously reported by others in China (Fu et al., 2018; Ha et al., 2018; Wen et al., 2018; Fang et al., 2019). However, it is noteworthy that such research was carried out prior to the appearance of African swine fever in China. This suggests that the infection of PCV3 in pigs results from using porcine plasma protein or contamination of feed raw materials that might be more strictly controlled following the appearance of African swine fever. The use of porcine plasma protein is prohibited by national regulations, and feed is produced at high temperatures during different periods (Trudeau et al., 2017). Therefore, we speculated that PCV3 might not be widely prevalent in some regions of China due to the current pressure of African swine fever. Nonetheless, a more comprehensive evaluation is needed, particularly in Hebei and Fujian provinces, where numerous cases have been reported. Thus, providing a more thorough and precise basic databank could help reveal the underlying cause(s) of PCV3 transmission or infection in large-scale pig farms in China. In this study, the results revealed that the overall positive rate of PCV2 in some regions of China was 24.04% (3,637/15,130). Specifically, PCV2 showed positive rates of 25.61% (599/2,344), 33.64% (529/1,801), 16.79% (391/2,329), 8.31% (133/1,600), 20.30% (307/1,512), 22.63% (415/1,834), 42.36% (726/1,799), and 26.22% (501/1,911) in Hubei, Hunan, Henan, Jiangxi, Shanxi, Guangdong, Hainan, and Heilongjiang provinces, respectively. Therefore, PCV2 infections exist, to a varying extent, in these regions of China, with notable regional differences. Hainan province was affected the most (42.36%). In general, the differences appear to be linked to feeding density, minimal environment control, biosafety levels, feeding management protocols as well as disease prevention and control procedures of the pig farms; geographical environment and natural climate may also be influencing factors (Lim et al., 2011).

### 4.3. The influence of geographical location on PCV prevalence

Hainan province is an island located in the southernmost region of China. The high infection rate (42.36%) of PCV2 in Hainan province could be attributed to intensive pig farms with insect vectors that frequently spread viral diseases, the open design of production and breeding buildings, and the large temperature differences between day and night. While Jiangxi province, located in the central China, has a low positive rate of PCV2 (8.31%). These results indicate that due to the pressures of African swine fever, changes in the pig industry promoted the transformation and upgrading of large-scale pig breeding facilities and equipment. In addition, biosafety-related facility upgrades and awareness increased. Finally, improved and refined health and production management to an even greater extent took place. Thus, all of these changes could have reduced the spread of PCV2.

### 4.4. The transmission pathways of PCV2

The surveillance results from the eight provinces revealed that compared with the numbers observed after African swine fever first appeared, the positive rate of PCV2 was higher prior to the appearance of African swine fever. This result provisionally suggests that biosafety measures, upgrading facilities, and reductions in pig population density might decrease the horizontal transmission of PCV and could contribute to the lower positive rate of PCV2 in Jiangxi province compared to the other provinces. However, gilts, primiparous sows, multiparous sows, lactating pigs, nursing pigs, and fattening pigs exhibited a distinct upward trend in their PCV2-carrying rate. Overall, the PCV2-carrying rate was significantly lower in sows and suckling piglets than that observed in nursing and fattening pigs. Both the vertical transmission in pigs and the horizontal transmission between pigs were present.

Furthermore, the excretion rates for PCV2 in pigs in the presence of African swine fever were systematically analyzed in this study. The overall data from the eight provinces indicated that despite the gradual upgrades made in biosafety measures, the virus was still excreted *via* different pathways. Colostrum showed a mean excretion rate of 26.65% for PCV. The fact that the virus can be excreted from the colostrum implies that infection of the digestive tract may occur in suckling pigs in the delivery room. As the infection becomes progressively more severe, some suckling pigs, after weaning (or in the delivery room), will suffer from viremia, PMWS, or even acute and chronic death in severe cases. In addition, there may be mixed infections present with epidemic diarrhea, which produces delivery room diarrhea syndrome (Jung et al., 2006; Opriessnig et al., 2011; Tzika, 2017), which consequently causes severe losses to farms.

Notably, infected sows may not be viremic, but their colostrum can still contain virus, which suggests that the virus may enter the mammary glands *via* other body fluids or pathways (Gerber et al., 2011). In this study, the results showed that the anus (25.30%) and colostrum (26.65%) were comparable with respect to the excretion rate for PCV, indicating that circovirus can be excreted through the feces and also suggesting that the digestive tract is vulnerable to circovirus. Thus, the gastrointestinal infection pathway has been elucidated *via* colostrum and anal excretion for PCV, which emphasizes the importance of horizontal transmission in certain circumstances (Patterson and Opriessnig, 2010; Rose et al., 2012).

The circovirus detected in nasal swabs partially reflects the horizontal transmission ability of the virus in the respiratory tract. Due to the presence of African swine fever, a potential downward trend was observed in the horizontal transmission ability of circovirus in the respiratory tract. The positive rate of nasal swabs (12.81%) was lower than that of vulvar swabs (15.47%), indicating that the horizontal transmission ability of circovirus in the respiratory tract was lower than in the reproductive tract and much lower than in the digestive tract, which is inconsistent with our traditional understanding (Rose et al., 2012).

Furthermore, it is important to note that the overall circovirus-carrying rate in semen was relatively high (22.71%), greatly affecting the ability to control viral infection when breeding. If a negative pathogen outcome cannot be guaranteed in semen, the reproductive system of sows bred with circovirus-positive semen may become readily infected. The fact that a boar may be mated with 50 or more sows further expands the infection capacity with the potential to cause an explosive spread of PCV2. Concerning the comparison of horizontal transmission, the data obtained from specific background values systemically made at the early warning level revealed that the infection rate of PCV2 was relatively high in regions with high pathogen-carrying rates in semen, and the opposite results occurred in areas with a low semen pathogen-carrying rate.

### 4.5. Analysis of clinical signs in pigs infected with PCV2

The issues of how many pigs were affected clinically by PCV2 under the pressure of African swine fever, as well as whether the numbers changed before and after the initial reports of African swine fever, were explored in this study. The results revealed that the highest positive rate was detected in afterbirth (41.67%), and a lower positive rate was discovered in offspring pigs. Therefore, testing positive for the virus in the afterbirth is not equivalent to offspring infection, and appropriate prevention and control strategies are able to block the passage of the afterbirth virus infection to offspring pigs. This might be explained by the idea that the high afterbirth detection rate might be related to insemination with virus-positive semen, allowing the pathogen to settle and multiply in the reproductive system after the reproductive tract was infected *via* the semen (Gava et al., 2008; Grasland et al., 2008; Dissertations and Gradworks, 2009; Opriessnig et al., 2011).

The kidney also showed a high positive rate for PCV2, which is consistent with previous reports (Kleymann et al., 2020), and high levels of PCV can induce PDNS. In addition, a high positive rate also was observed in immune organs such as the liver, spleen, and lymph nodes, which is associated with the frequent invasion of immune organs by PCV2 (Shi et al., 2021), and viremia may occur in severe cases (Correa-Fiz et al., 2020; Lopez-Lorenzo et al., 2021). PCV2 also was detected in the intestines and stomach, which is indicative of gastrointestinal infection with circovirus, and agrees with recent reports (Liu et al., 2019; Yang et al., 2020). The relatively high positive rate observed in the coronary sulcus tissues is basically identical to clinical observations, which helps provide evidence of sudden unexplained death in some pigs (Mikami et al., 2005). Nonetheless, there have been no previous reports concerning this condition thus far.

PCV2-positive outcomes occurred in cerebrospinal fluid, and research into PCV2 invasion of brain tissues has been reported in the literature, but the mechanism of action is unclear (Tummaruk and Pearodwong, 2016). Circovirus detection suggested that after the appearance of African swine fever, a series of comprehensive prevention and control measures (e.g., biosafety, disease prevention and control methods, closed management of pigs, and frequent disinfection of pigs) formulated by farms might cause variations in levels of circovirus, facilitate the ability of the pathogen to break through the blood–brain barrier. This variation might enhance pathogen virulence, posing severe threats to pigs, and deserves closer attention.

To further elucidate circovirus-induced viremia in pigs with clinical signs and moribund states or reproductive disorders, blood samples were collected before on-site necropsy for viremia detection. The results revealed that the detection rate was 27.81% (223/802) in all pigs. This suggested a relatively high incidence rate of circovirus-induced viremia in pigs with clinical signs and in a moribund state at the pig farms. Thus, under the novel pressures encountered by the presence of African swine fever, the ability of circovirus to induce viremia might be enhanced. In this study, systematic analysis was carried out on pigs with clinical signs and those prone to viremia. The results confirmed that the highest detection rate of viremia was discovered in nursing and fattening pigs with red and cyanotic skin and the appearance of a sudden moribund state, yielding a 58.79% (97/165) infection rate. Thus, it may be concluded that PCV2-induced viremia could be the primary cause of red and cyanotic skin and sudden death in nursing and fattening pigs.

Among nursing pigs and sows with emaciation, loss of appetite, and low-grade fever, those with chronic subclinical signs that turned into an acute moribund state showed a viremia rate of up to 22.09% (19/86), suggesting that circovirus-induced viremia might be one of the causes of death of the affected pigs. Among suckling piglets, moribund weak piglets showed a relatively high detection rate of viremia, which may be attributed to congenital infection with circovirus, oral infection (including from colostrum), infection from polluted environments, and accumulation of virus load. Among fattening pigs, the relatively high detection rate of viremia was found in diseased pigs that died. The reason might be because chronic pathogen infections lead to reduced resistance in the diseased pigs and increase in the carrying rate of PCV in infected tissues. Additionally, diarrhea might occur when the accumulative infection load is excessive in the digestive tract or when mixed infections occur, similar to the porcine epidemic diarrhea virus (PEDV), leading to dehydration or multiple organ failure and resulting in death.

Stiff piglets that appeared among nursing piglets or piglets that died of encephalitis showed a relatively lower detection rate of viremia, suggesting that stiff piglets may have some tolerance to viremia, and after the viremia disappears, they die of organ failure (Opriessnig and Langohr, 2013). Also, the detection rate of encephalitis-induced viremia was unexpectedly low, while the detection rate in cerebrospinal fluid was much higher than that of encephalitis-induced viremia, indicating that PCV2 possibly infects pigs by breaking through the meningeal barrier but not through the blood circulation.

Prior to the reports of African swine fever in China, PCV2 was often found in combination with several other pathogens (e.g., PRRSV, PRV, CSFV, and PEDV), producing mixed infections. Since African swine fever appeared, reports have indicated extremely low single infection rates of PCV2 [1.91% (289/15,130)]. Notably, mixed infections were predominant in the present study, particularly PCV2 with PRRSV, which is consistent with current reports. However, the proportion of mixed infections has shown a downward trend, and the double infection rate is 10.63% currently. Furthermore, some changes have taken place in the types of mixed infections that are observed. For example, there was a high proportion of mixed infections with PPV, especially double mixed infections with PCV (2.08%). This might be attributed to the situation that when the market for pig industry flourished, the prevention of PPV in numerous gilts was not systematic, and production was carried out without necessary and sufficient immunization. Compared with the reports in Shandong and Hebei provinces, PEDV also showed a notably lower rate of mixed infections (0.96%), revealing the drastically declining incidence of diarrhea under the influence of the industry’s general emphasis on biosafety.

### 4.6. Sequential evolution of PCV2 in eight provinces of China

In addition, in the present study, the positive samples were sequenced and analyzed to elucidate the prevalence of PCV2 strains in some regions in China. In this study, the homology was 95.36 to 100% between the 24 isolates and 95.46 to 99.98% between the isolates and the foreign reference strains. Therefore, the overall variation range in this study was small, which was inconsistent with the published literature (Park et al., 2013). The results of gene clustering showed that nine strains belonged to PCV2a, seven strains belonged to PCV2b, two strains belonged to PCV2c, and six strains belonged to PCV2d. However, PCV2e was not detected at all in the study, although it has been detected in lung samples in pigs in Guangdong and Jiangxi (Liu et al., 2018; Xu et al., 2022) Overall, the circovirus epidemic gene clusters varied between the provinces, showing regional characteristics, which might be correlated with the provenance and breeding environment in the different provinces, as well as improvements in biosafety systems. Due to the presence of African swine fever, the interaction between pigs among different provinces has declined, and closed-loop production has been emphasized.

A note of caution should be made that PCV2c appeared in Hunan and Heilongjiang provinces which had not been documented previously. The pigs testing positive for PCV2c were all Danish pigs, suggesting that PCV2c may have been introduced *via* importation into China since the prevalence of PCV2c has been reported in Europe for a long time (Xiao et al., 2015). These conditions make the prevention and control of circovirus more complex.

According to previous reports, there have been many PCV2 genotypes in China, with PCV2a, PCV2b, and PCV2d as the predominant genotypes (Guo et al., 2010; Zhao et al., 2010; Wei et al., 2012; Huang et al., 2021). However, the new gene cluster PCV2c must be noted by the industry. Variations in the PCV2 Cap protein allow for changes in viral immunogenicity and cell tropism, leading to variations in virulence. The analysis of the amino acid sequences of Cap revealed several amino acid variations in the Cap protein. Based on epidemiological investigations, four gene clusters, PCV2a, PCV2b, PCV2c, and PCV2d (Xiao et al., 2015; Franzo et al., 2016), were detected *as per* the gene-specific locus standard (Wei et al., 2013; Wu et al., 2019), with loci 86 to 91 as the specific sequence loci for Cap protein typing: TNKISI in PCV2a, SNPRSV in PCV2b, EGAQTP in PCV2c, and SLPNTV in PCV2d. PCV2e was not detected, which was inconsistent with the results presented in other studies (Liu et al., 2018; Tsai et al., 2019; Kang et al., 2022).

In addition, the molecular epidemiological investigation showed that new amino acid mutations were detected. For instance, the loci 70(D), 73(R), and 178(N) in PCV2a were separately replaced by G (2)/L(1)/T(1), N(3)/*F*(2), and R(2), which was different from the mutations of 73(R) to (L) and 178(N) to S(1) reported by Wei et al. (2013). In PCV2b, 57(I) and 210(E) served as specific loci in addition to the basic specific loci 86–91 (Wei et al., 2013). This study showed that 178(N) mutated to R(4), which was inconsistent with the mutation of 178(N) to D(1) described in another report (Wen-fang et al., 2020), and there were no mutations at these two amino acid positions.

The locus 70(D) of PCV2c was deleted in the sequences of the two strains isolated in this study, which has not been reported in other studies (Xiao et al., 2015; Franzo et al., 2016; Liu et al., 2016). In PCV2d, besides the above-mentioned specific loci, 53(I), 68(N), 215(I), and 234(K) also acted as specific loci (Wen-fang et al., 2020), and these amino acid positions were not mutated. Moreover, the amino acid mutations in PCV2a, PCV2b, PCV2c, and PCV2d, shown in Table 4, have not been reported in the literature as being related to PCV2. Notably, the unique glycosylation locus 143–145 (NYS) of the Cap protein experienced point mutations at 145 (S) in this study. Mutations were observed in the amino acid sequences in some of the strains, which are possibly implicated in virulence differences between the different strains. These results had not been reported in the relevant PCV2 literature prior to the onset of African swine fever, possibly implying that PCV2 exhibits more variability after the onset of African swine fever. The above amino acid loci were highly variable, allowing the amino acids to change from hydrophobic to hydrophilic, with the potential to cause changes in the antigenicity of the PCV strains. Furthermore, whether the variation of these antigens is correlated with the enhanced ability of PCV2 to break through the meningeal barrier, thus inducing viremia as well as the emergence of novel gene cluster PCV2c, needs to be investigated and validated in future research.

## 5. Conclusion

Two reasons may explain the increase of porcine circovirus disease in China. First, the prevention and control measures of PCV are weakened due to the prevention and control of African swine fever. Second, the biosafety facilities in some pig farms are backward due to the different geographical location and economic development. In addition, we found that PCV2a, pcv2b, PCV2c, and PCV2d are still prevalent in Hubei, Hunan, Henan, Jiangxi, Shanxi, Guangdong, Hainan, and Heilongjiang provinces of China, and can be transmitted both horizontally and vertically.

## Data availability statement

The original contributions presented in the study are included in the article/supplementary material, further inquiries can be directed to the corresponding authors.

## Author contributions

CL, GL, and KT provided conceptualization, methodology, writing, reviewing, and editing. XT, CL, and YW provided investigation and data analysis. JY and TL provided methodology and investigation. KT and TL provided visualization. GL and LG provided project administration and manuscript checking. XY and JZ provided supervision and visualization. All authors contributed to the manuscript and approved the submitted version.
