# Erratum: Pathogenic ecological characteristics of PCV2 in large-scale pig farms in China affected by African swine fever in the surroundings from 2018 to 2021

**DOI:** 10.3389/fmicb.2023.1345300

**Published:** 2023-12-08

**Authors:** Frontiers Production Office

**Affiliations:** Frontiers Media SA, Lausanne, Switzerland

**Keywords:** PCV2, PCV3, dual nested PCR, molecular epidemiology, positive rate of blood samples, excretion rate for PCV

Due to a production error there was a mistake in Table 2 and Table 3 as published. Table 2 and Table 3 were published in the wrong order. The corrected order of Table 2 and Table 3 appears below.

**Table 2:** Distribution of different viremias.

**Sample type**	**Age stage**	**Clinical signs**	**Tested samples**	**Positive samples**	**Positive rate**
Whole blood	Suckling piglets	Weak piglet death	129	36	27.91%
	Suckling and nursing piglets	Piglet diarrhea	91	12	13.19%
	Nursing piglets	Piglets died of encephalitis	51	1	1.96%
	Nursing piglets	Dead stiff pig	60	1	1.67%
	Nursing and fattening pigs	Dead pigs with red and cyanotic skin	165	97	58.79%
	Fattening pigs	Residual pig death	54	9	16.67%
	Nursing piglets and sow	Emaciation and loss of appetite	86	19	22.09%
	Pregnant sows	Reproductive disorder	166	48	28.92%

Collection of blood from pigs with different clinical signs in pig farms for PCV2 etiological monitoring.

**Table 3:** Distribution of PCV2 in different organs.

**Organ type**	**Number of samples**	**Positive number**	**Positive rate**
Heart	71	10	14.08%
Cerebrospinal fluid	36	4	11.11%
Intestines	66	9	13.64%
Lung	71	9	12.68%
Liver	71	17	23.94%
Spleen	71	16	22.54%
Kidney	61	21	34.43%
Afterbirth	12	5	41.67%
Stomach	71	6	8.45%
Lymph gland	19	6	31.58%
Blood	245	50	20.41%

A total of 794 clinically suspected samples were necropsied, and the overall positive rate of PCV2 was 19.43%. PCV2 was detected in all tissue and organ types submitted for inspection.

The publisher apologizes for this mistake. The original article has been updated.

