# Supplementary material for: Pathogenic ecological characteristics of PCV2 in large-scale pig farms in China affected by African swine fever in the surroundings from 2018 to 2021
Source: Front Microbiol. 2023 Jan 4;13:1013617. doi: 10.3389/fmicb.2022.1013617 (PMC9845725; doi:10.3389/fmicb.2022.1013617)
Supplement: Supplementary file 1 [file Data_Sheet_1.zip › Tables.docx]

**Table S1:** The primer sequences for amplifying glycoprotein

| **Traget gene** | **Fragments** | **Primer sequence(5′–3′)** | **position** | **Fragment length/bp** | **TM** |
| --- | --- | --- | --- | --- | --- |
| cap protien | PCV2-O | F：CATCTTCAACACCCGCCTCT | 574-555 | 518 | 57.7 |
|  |  | R：GGATATTGTATTCCTGGTCGTAT | 63-85 |  | 51 |
|  | PCV2-I | F：GGGCGGTGGACATGATGAGAT | 503-483 | 254 | 60.1 |
|  |  | R：GGTTATGGTATGGCGGGAGGA | 250-270 |  | 59.4 |
|  | PCV3-O | F：GTGTGAGTGGATATACCGGGCAGTG |  | 1091 | 61.9 |
|  |  | R：ACCCACCCACCCAATAAATAC |  |  | 54.6 |
|  | PCV3-I | F：AGTGTTTTG ATGCCGC AGG AC |  | 902 | 57.7 |
|  |  | R：AAGGCCTCCAACTTTCCACGAAT |  |  | 59.1 |

Note: PCV2-O and PCV3-O were the primers in the first round of nested PCR; PCV2-I and PCV3-I were the primers in the second round of nested PCR. The TM was the optimum annealing temperature for the primers.

**Table S2:** The optimum reaction conditions for the nested PCR

| **Progress** | **Program** | **Temperature** | **Response time** | **Cycle** |
| --- | --- | --- | --- | --- |
| First round of reaction conditions | Predegeneration | 94 ℃ | 5 min | 1cycle |
|  | denaturation | 94 ℃ | 30 s | 35cycle |
|  | anneal | 56℃ | 30 s |  |
|  | Extend | 72 ℃ | 45 s |  |
|  | Final extend | 72 ℃ | 5 min | 1cycle |
|  | Save | 4 ℃ | N.A | N.A |
| Second round of reaction conditions | Predegeneration | 94 ℃ | 5 min | 1cycle |
|  | denaturation | 94 ℃ | 30 s | 35cycle |
|  | anneal | 58℃ | 30 s |  |
|  | Extend | 72 ℃ | 45 s |  |
|  | Final extend | 72 ℃ | 5 min | 1cycle |
|  | Save | 4 ℃ | N.A | N.A |

**Note:** N.A. stands for null or meaningless value.

**Table S3:** Comparison of four PCV2 and PCV3 detection methods

| **Sample type** | **Test method** | **Sample quantity** | **PCV2 positive rate** | **PCV3 positive rate** | **conformity** |
| --- | --- | --- | --- | --- | --- |
| Serum | Nest PCR | 150 | 68.67% | 0% | 100% |
|  | PCR | 150 | 61.33% | 0% | 89.32% |
|  | ELISA | 150 | 50% | 1% | 99% |
|  | qPCR | 150 | 68% | 2% | 98% |
| Genome-wide positive controls for PCV2 and PCV3 with a copy number of 10^5^-10^0^ | Nest PCR | 6 | 100% | 100% | 100% |
|  | PCR | 6 | 50% | 50% | 50% |
|  | qPCR | 6 | 83% | 83.33% | 83.33% |

Note: With a copy number of 10^5^-10^0^ PCV2 and PCV3 genome-wide positive controls for replicate experiments, each sample was replicated three times, and the replication rate was 100%. The detection rate of q-PCR was 2%, and the homology analysis and sequencing results showed false positives.

**Table S4:** The partial Pearson correlation between ASFV and PCV2

|  | **PCV2** | **ASFV** |
| --- | --- | --- |
| **PCV2** | . | 0.6014 |
| **ASFV** | 0.6014 | . |

Note: The partial Pearson correlation coefficient of PCV2 and ASFV was calculated between the residual obtained by linear regression of PCV2 and province and the residual obtained by linear regression of ASFV and province. After adjusting for the effect of provinces, the partial Pearson correlation between ASFV and PCV2 reached 0.6, indicating better correlation. All P values are less than 0.0001.
