# Supplementary material for: Pathogenic ecological characteristics of PCV2 in large-scale pig farms in China affected by African swine fever in the surroundings from 2018 to 2021
Source: Front Microbiol. 2023 Jan 4;13:1013617. doi: 10.3389/fmicb.2022.1013617 (PMC9845725; doi:10.3389/fmicb.2022.1013617)
Supplement: Supplementary file 1 [file Data_Sheet_1.zip › Figure legends.docx]

Figure S1. Establishment of nested PCR method for pcv2 and pcv3

(A). Double nested PCR specificity test

①Results of PCV2 nested PCR lateral specificity test ③Results of PCV3 nested PCR lateral specificity test

②Results of PCV2 nested PCR with medial specificity test ④Results of PCV2 nested PCR with medial specificity test

PS：M：DL2000 DNA Marker；①-②1:PCV2;③-④1:pcv3； ①-④：2-7：CSFV, PPV, PRV, PRRSV, E.coli, Staphylococcus aureus；8：Negative control；9：Reagent contrast

(B). Nested PCR sensitivity test

①PCV2, PCV3 lateral sensitivity test results

②PCV2, PCV3 inner sensitivity test results

PS：M：DL2000 DNA Marker；In 1-10, the template dilution gradient is10-1,10-2, 10-3, 10-4, 10-5, 10-6, 10-7, 10-8, 10-9, 10-10.

(C). Nested PCR interference test

①PCV2 lateral interference test results ③PCV3lateral interference test results

②PCV2 inner interference test results ④PCV3inner interference test results

PS：M：DL2000 DNA Marker；1-4 Mix genome；5：Negative control；6：Reagent contrast

Figure S2. Nucleotide alignment and the phylogenetic analysis based on the cap genes of PCV2.

(A). Nucleotide alignment of the Cap gene. Comparison of the nucleotide and capsid (Cap) among the 24 isolates and the ten classical sequences. Gene nucleotide sequences of Cap were aligned by DNAStar. b. The phylogenetic analysis based on the cap genes of PCV2.on the cap genes of 24 PCV2 strains, constructed using MEGA 5.0 software. “Filled circles” represent the resulting sequence of PCV2. No markers represent classical reference strains of different PCV2 genotypes. The phylogenetic tree was constructed using the neighbor-joining method in MEGA 5.0 software with a bootstrap test of 500 replicates.
