# Supplementary figures and images for: Pathogenic ecological characteristics of PCV2 in large-scale pig farms in China affected by African swine fever in the surroundings from 2018 to 2021
Source: Front Microbiol. 2023 Jan 4;13:1013617. doi: 10.3389/fmicb.2022.1013617 (PMC9845725; doi:10.3389/fmicb.2022.1013617)

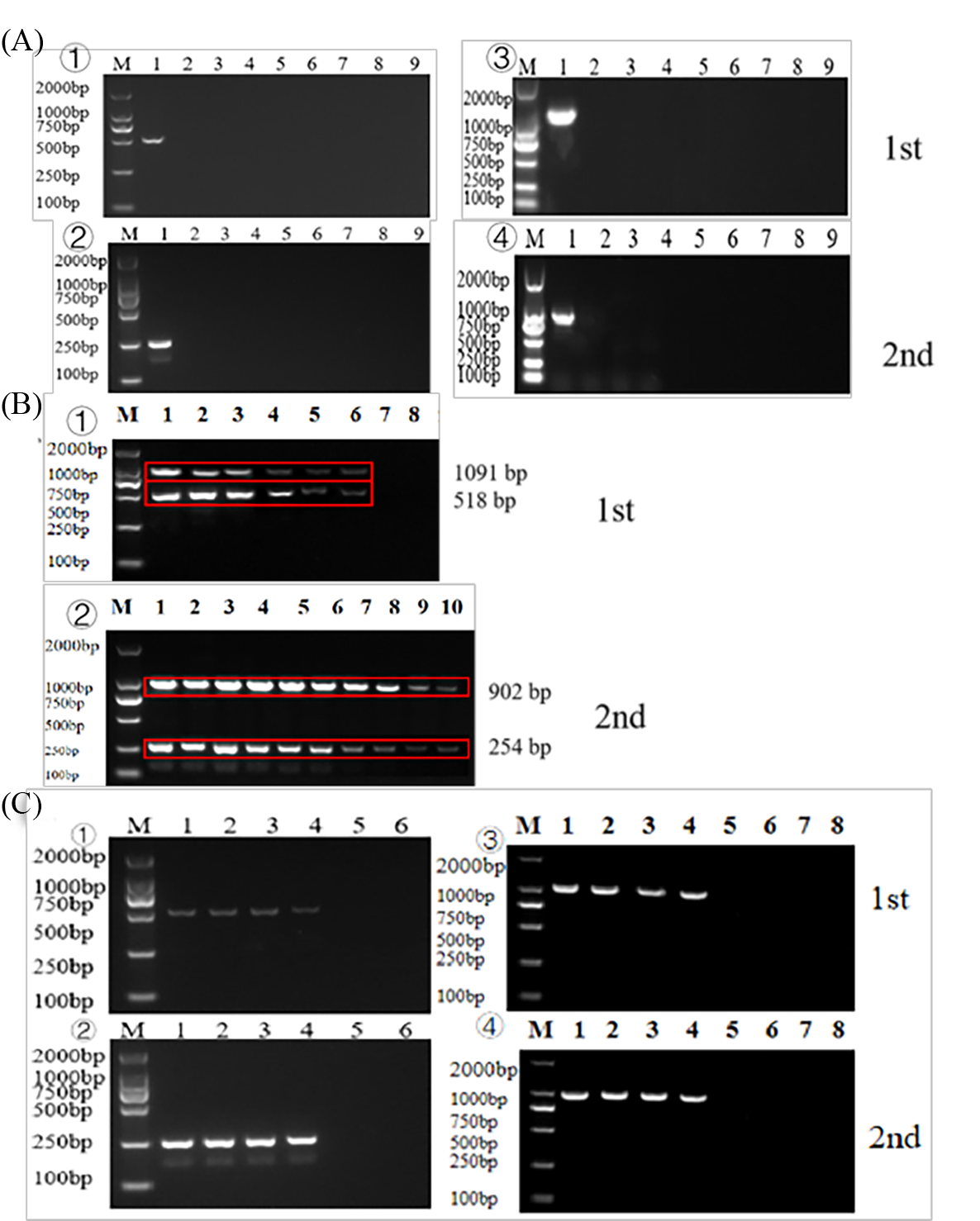

Supplement: Supplementary file 1 [file Data_Sheet_1.zip › Fig S1.tif]

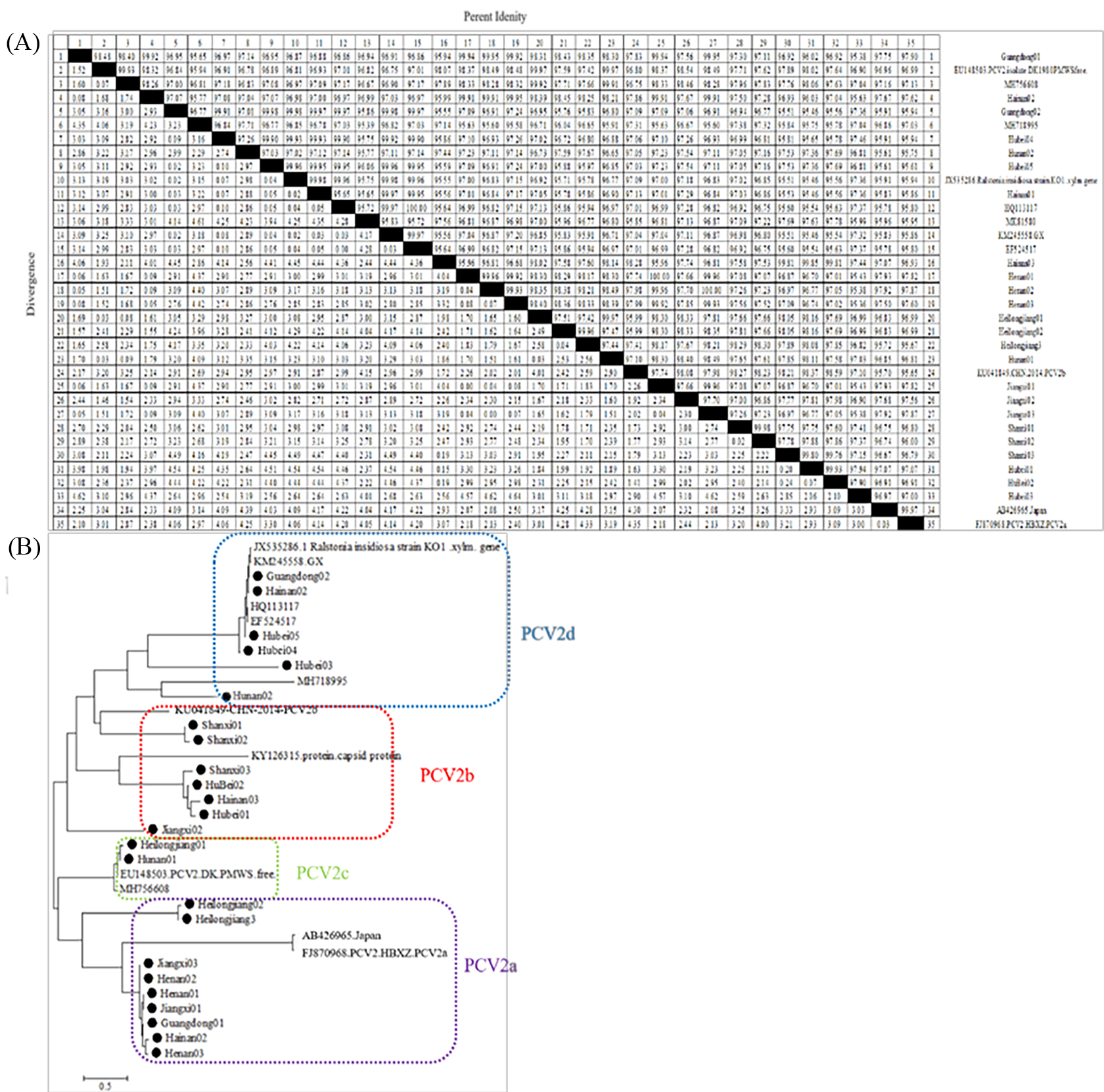

Supplement: Supplementary file 1 [file Data_Sheet_1.zip › Fig S2.tif]
